# Phylogenetic Triage and Risk Assessment: How to Predict Emerging Phytoplasma Diseases

**DOI:** 10.3390/biology12050732

**Published:** 2023-05-17

**Authors:** Katrin Janik, Bernd Panassiti, Christine Kerschbamer, Johannes Burmeister, Valeria Trivellone

**Affiliations:** 1 Laimburg Research Centre, Functional Genomics, Laimburg 6-Pfatten (Vadena), 39040 Auer, South Tyrol, Italy; katrin.janik@laimburg.it (K.J.); christine.kerschbamer@laimburg.it (C.K.); 2 Independent Researcher, D-81543 Munich, Germany; 3 Institute for Organic Farming, Soil and Resource Management, Bavarian State Research Center for Agriculture, 85354 Freising, Germany; johannes.burmeister@lfl.bayern.de; 4 Illinois Natural History Survey, Prairie Research Institute, University of Illinois at Urbana-Champaign, Champaign, IL 61820, USA

**Keywords:** archives, bio-inventories, aster yellow, Bavaria, DAMA protocol, geographic distribution, metabarcoding, phytopathogens, risk evaluation, risk heat map

## Abstract

**Simple Summary:**

Phytoplasma diseases are a major threat to economically important crops and are usually only managed after the disease has occurred. In this study, the presence of two phytoplasmas in the aster yellows group were detected in insect samples collected from different agricultural settings in South Germany during a biodiversity survey. We used these findings to apply a proactive document-assess-monitor-act (DAMA) protocol to assess the potential for phytoplasma disease outbreaks in croplands in Bavaria, Germany. Notably, we carried out a phylogenetic triage and assessment to generate a risk heat map to select a minimum of seven leafhopper species that may serve as insect vectors of aster yellow phytoplasmas. To detect the presence of aster yellow phytoplasma in susceptible crops (e.g., wheat and barley) in Bavaria, we suggest specific monitoring activities and screening of these insect species as a proactive measure. This is the first time the DAMA protocol is applied in the field of phytopathology.

**Abstract:**

Phytoplasma diseases pose a substantial threat to diverse crops of agricultural importance. Management measures are usually implemented only after the disease has already occurred. Early detection of such phytopathogens, prior to disease outbreak, has rarely been attempted, but would be highly beneficial for phytosanitary risk assessment, disease prevention and mitigation. In this study, we present the implementation of a recently proposed proactive disease management protocol (DAMA: Document, Assess, Monitor, Act) for a group of vector-borne phytopathogens. We used insect samples collected during a recent biomonitoring program in southern Germany to screen for the presence of phytoplasmas. Insects were collected with malaise traps in different agricultural settings. DNA was extracted from these mass trap samples and subjected to PCR-based phytoplasma detection and mitochondrial cytochrome c oxidase subunit I (COI) metabarcoding. Phytoplasma DNA was detected in two out of the 152 insect samples analyzed. Phytoplasma identification was performed using iPhyClassifier based on 16S rRNA gene sequence and the detected phytoplasmas were assigned to ‘*Candidatus* Phytoplasma asteris’-related strains. Insect species in the sample were identified by DNA metabarcoding. By using established databases, checklists, and archives, we documented historical associations and records of phytoplasmas and its hosts in the study region. For the assessment in the DAMA protocol, phylogenetic triage was performed in order to determine the risk for tri-trophic interactions (plant–insect–phytoplasma) and associated disease outbreaks in the study region. A phylogenetic heat map constitutes the basis for risk assessment and was used here to identify a minimum number of seven leafhopper species suggested to be monitored by stakeholders in this region. A proactive stance in monitoring changing patterns of association between hosts and pathogens can be a cornerstone in capabilities to prevent future phytoplasma disease outbreaks. To the best of our knowledge, this is the first time that the DAMA protocol has been applied in the field of phytopathology and vector-borne plant diseases.

## 1. Introduction

The increased number of plant disease outbreaks worldwide accentuates the urgent need for proactive approaches based on effective and timely risk assessment and management [1,2]. Plant pathogens have traditionally been considered to be locked in an evolutionary “dead end” relationship with their hosts, i.e., that they establish highly specialized associations with a few host species over time such that if the host goes extinct, the pathogen also goes extinct (reviewed in [3]). Under this paradigm, emergence of a new infectious plant disease (EIPD) was considered as a rare and unpredictable event. Building on this understanding of pathogen–host interactions, approaches to control new EIPDs have been reactive or response-based. Linked to preparation and palliation of such reactive pathways, largely waiting for a pathogen to emerge, have been shown to be unsuccessful and unsustainably expensive [4,5]. In contrast, the Stockholm Paradigm (SP), recasting the dynamics of symbiotic associations in the biosphere, was proposed to explain the ecological and evolutionary processes driving the emergence of new host–pathogen associations [6]. Concurrently, the SP served as a conceptual foundation for implementation of an operational extension for grounded, actionable, evidence-driven policy, the DAMA (Document, Assess, Monitor, Act) protocol (reviewed in [7,8]). DAMA is the first comprehensive and integrated proposal for a proactive stance and capacity to anticipate and mitigate emerging pathogens and diseases [9]. The DAMA protocol can be also integrated into use-inspired research programs to produce meaningful information which facilitates and expedites fact-based decision making.

The DAMA protocol was originally articulated in the context of animal pathogens and disease and fully applied for the first time to zoonotic diseases, focused on early detection of pathogens with evaluation of diverse assemblages of mammalian and arthropod hosts [10,11,12]. Establishing the universality of SP and DAMA across the biosphere for a range of natural and managed habitats encompassing animal and plant systems is essential. However, ongoing impacts of phytopathogens on agricultural economies and global food security also highlight a crucial need to implement the DAMA protocol for plant pathogens [2,7,13,14].

Phytoplasmas are economically important plant pathogens associated with a wide spectrum of degenerative symptoms affecting several annual and perennial crops, bushes, fruit and ornamental trees and native plants and can lead to massive yield loss in economically important crops [1,15]. Phytoplasmas are transmitted plant-to-plant by phloem-feeding insects, mainly leafhoppers, planthoppers, and psyllids [16]. 

Recent studies revealed that screening potential insect vectors is an efficient alternative method for discovering and characterizing host–phytoplasma associations, allowing the presence of phytoplasmas to be detected in the environment in the absence of a disease outbreak [17,18,19]. By using existing data from different biorepositories, this promising strategy focuses on the evaluation of the entire suite of potential insect vectors and plant hosts present in a defined macro-area. Taking into account the important role of vectors in spreading phytoplasmas across habitat interfaces, the risk of disease outbreaks can be assessed by evaluating the phylogenetic relatedness among host lineages and the opportunity for new encounters between pathogens and potential hosts (colonization processes) to yield previously undocumented associations. The capability of pathogens to survive in suboptimal hosts allows them to expand their “fitness space” across the landscape [20,21]. These dynamics demonstrate the potential power of the DAMA protocol applied at regional or local levels, for phytoplasmas and a broader array of plant pathogens.

In this study, we used historical data and recent bio-inventories to apply the DAMA protocol for the first time to a plant patho-system (phytoplasmas and their hosts) with the aim of providing actionable information for risk assessment of potential phytoplasma outbreaks and risk management in the Bavarian region, southern Germany.

## 2. Materials and Methods

### 2.1. Study Regions and Insect Collections

**Figure 1:**
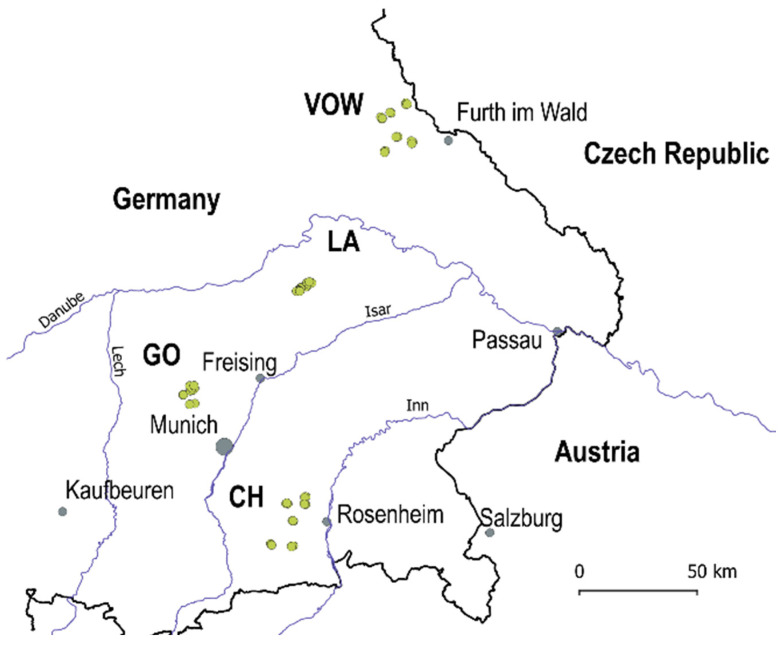
Study area includes four macro-areas in the Bavarian region (south Germany): Upper Palatine Forest (VOW), Kelheim—Laaber (LA), Dachau—Glonn (GO) and Chiemgau (CH). Dots in green are the 56 locations surveyed in 2020.

In 2020, a Regional Insect Inventory (hereafter RII 2020) investigating the influence of agri-environmental measures on insect diversity and biomass was launched to evaluate the insect communities in crop fields and grasslands located in the Bavarian region, southern Germany. In total, 56 locations in four macro-areas were surveyed: Upper Palatine Forest (VOW, 18 locations), Kelheim—Laaber (LA, 10 locations), Dachau—Glonn (GO, 10 locations) and Chiemgau (CH, 18 locations) (Figure 1, green dots). The locations in VOW and CH are grasslands, and the landscapes are dominated by other grasslands. The locations in LA and GO are crop fields. Macro-areas LA and GO are strongly transformed agricultural landscapes with a few hedgerows (i.e., ecological compensation areas) and woody patches surrounding the fields. The average distance among locations was about 11,600 m in grassland macro-areas CH and VOW and 3600 m for agricultural macro-areas GO and LA.

Insect collection was carried out using malaise traps, a sampling method that has been successfully applied in various ecological studies [22,23,24] and proven to be feasible for collecting a broad spectrum of insects from different trophic guilds [25]. Standardized malaise traps of the “Bartak” type, provided by Bioform [26], were used during the RII 2020 survey. In grassland macro-areas (VOW and CH), one malaise trap (hereafter: sampling site) was deployed in each grassland unit for a total of 36 sampling sites. To account for landscape heterogeneity in agricultural macro-areas (LA and GO), two malaise traps were deployed at each location, one at a distance of five m apart from the border of the field (W trap) and another one in the center of the field (M trap). The minimum distance between the two malaise traps was 80 m. A total of 40 sampling sites were surveyed. Field crops were mainly winter cereals (15/20) and occasionally maize (2/20), spring cereals (1/20), sugar beet (1/20) and rapeseed (1/20) in the field center. In 13 sites the W trap was placed on a hedgerow.

The malaise traps were opened and active for two consecutive weeks in three periods: June, July and September. Each bulk insect sample, from each week, was separated into two fractions (macro and micro) based on insect size using a sieve with a 6 mm mesh width. Samples from one week were stored in 80% ethanol solution before DNA extraction. Samples from the other week were used for morphological identification and voucher specimens deposited at Illinois Natural History Survey (University of Illinois at Urbana-Champaign, USA).

### 2.2. DNA Extraction, Metabarcoding and Insect Identification

Dried and homogenized samples (FastPrep-96™) were subjected to tissue lysis in 10 mL of a premixed insect lysis buffer containing 10% of Proteinase K for eight hours at 56 °C. Genomic DNA was then extracted into 50 µL elution buffer AE following the manufacturer’s instructions of the “DNeasy Blood & Tissue” plate kit (Qiagen, Hilden, Germany). Amplification of the CO1-5P target region and PCR reactions were carried out using the MyTaqTM Plant-PCR Kit (Bioline GmbH, Luckenwalde, Germany) with Illumina-ready fusion primers derived from the primer pair dgLco, 5′-GGWACWGGWTGAACWGTWTAYCCYCC-3′; mlCOIntF, 5′-TAAACTTCAGGGTGACCAAARAAYCA-3′ published by Leray et al. [27]. Initially developed for marine fauna, this primer pair proved to be very effective on terrestrial arthropods [24,28]. We used the same PCR conditions as reported in Leray et al. [27] and Morinière et al. [28]. For all samples, amplification success and fragment length were checked using gel electrophoresis in a 1% TAE gel using GelRed (Genaxxon bioscience GmbH, Ulm, Germany). Amplified DNA was cleaned up and resuspended in 50 µL molecular water for each sample before proceeding.

Illumina Nextera XT (Illumina Inc., San Diego, CA, USA) indices were ligated to the samples in a second PCR reaction applying the same annealing temperature as for the first PCR reaction but with seven cycles. Ligation success for all samples was confirmed using gel electrophoresis (condition as above). DNA concentration was measured with a Fluoroskan plate reader (Life Technologies, Carlsbad, CA, USA) using the Qubit fluorometer dsHS chemicals (Life Technologies, Carlsbad, CA, USA), and samples were combined into pools containing equimolar concentrations of 100 ng each. These pools were purified, and size selected using Next-Generation Sequencing (NGS) magnetic beads (MagSi-NGSPrep Plus, Magtivio, Nuth, The Netherlands) for downstream sequencing applications. After another verification of DNA concentration and amplicon size using Qubit fluorometer (Life Technologies) and Bioanalyzer (Agilent Technologies, Santa Clara, CA, USA), the final library was compiled. The amplicon size of the final library followed a size distribution of ~520 bp. High-Throughput Sequencing (HTS) was carried out using an Illumina MiSeq with v3 (2 × 300 bp, 600 cycles, maximum of 25 Mio paired-end reads) chemistry.

Raw FASTQ files were combined, and sequence processing was performed with the VSEARCH v2.4.3 suite [29]. Cutadapt v1.14 [30] was used to screen for correct fusion primer adapter sequences and complete primer removal. All the sequenced samples yielded reverse reads of high enough quality to enable paired-end merging. Quality filtering was performed with the “fastq_filter” program of VSEARCH using the following options fastq_maxee = 2 and minimum bp length = 100. Sequences were dereplicated with “derep_fulllength”, first at the sample level, and then concatenated into one fasta file, which was then dereplicated. Chimeric sequences were removed using “uchime_denovo”. Remaining sequences were clustered into OTUs at 97% identity. To reduce likely false positives, a cleaning step was employed which excluded read counts in the OTU table of less than 0.01% of the total. OTUs were blasted against a custom database downloaded from Barcode of Life Data System (BOLD) [31]. Downloaded data included taxonomy and BIN information of Central European animals and processed by means of Geneious (v.10.2.5—Biomatters, Auckland, New Zealand), and following the methods described in Morinière et al. [28,32]. The OTU ID, BOLD Process ID, BIN, Hit-%-ID value (% of overlap similarity, identical base pairs, of an OTU query sequence with its closest counterpart in the database), length of the top BLAST hit sequence, phylum, class, order, family, genus, and species information for each detected OTU was exported from Geneious and combined with the OTU table generated by the bioinformatic pipeline. OTUs were then assigned to the respective BIN as described in Morinière et al. [32]. OTUs were then assigned to the respective BIN as described in Morinière et al. [28]. Additionally, the BOLD reference database was used to retrieve BIN species and BIN countries for every OUT, Next, Hit-%-IDs were aggregated over OTUs that found a hit in the same BIN and shown in the corresponding column as % range. To validate the BOLD BLAST results, a separate BLAST search was carried out in Geneious (using the same parameters) against a local copy of the National Center for Biotechnology Information (NCBI) [33]. Moreover, we applied an annotation of OTU sequences using a CO1-trained Ribosomal Database Project (RDP) classifier, a naïve Bayesian classifier, as described in Porter and Hajibabaei [34]. The resulting taxonomies from BOLD, NCBI databases and RDP classifier were then concatenated within a consensus taxonomy according to highest consensus in taxonomic overlap resulting in a consensus table. For each sample, hemipteran Auchenorrhyncha insects were selected for this study and the minimum number of genera using the “speciesMin” R-function by Burmeister and Panassiti [35] was calculated.

### 2.3. Phytoplasma Detection and Classification

We used an aliquot of the sample collected for screening of bacterial pathogens of phytoplasmatic origin. To analyze for presence of phytoplasmas in bulk insect samples, the micro fraction was selected because, among others, it includes insects known to be vectors of phytoplasmas in the suborder Auchenorrhyncha. For this screening we selected samples collected in the first and third period that cover the entire period of presence of different potential vector species. A total of 152 samples were analyzed from all four macro-areas. The analysis was performed by running a two-step nested PCR using the primers described in Gundersen and Lee [36]. The first PCR was run as follows: 0.1 µL dNTPs (40 mM, i.e., 10 mM each dNTP), 2.5 µL 10X DreamTaq Buffer and 0.5 µL DreamTaq Polymerase (5 U/µL) (both Thermo Scientific, Waltham, MA, USA) were combined with 1.3 µL of each of the primers R16mF2 (10 µM) and R16mR1 (10 µM) and nuclease-free water was added to reach a total volume of 23 µL. The insect pool DNA was diluted 1:100 in nuclease-free water and 2.0 µL of the diluted DNA solution (10–50 ng/µL) was added to the PCR master mix. Each PCR sample was subjected to the following thermal-cycling conditions: an initial 3 min DNA denaturation at 95 °C followed by 35 cycles of 30 s denaturation at 95 °C, 30 s annealing at 58 °C and elongation for 2 min at 72 °C. The amplification was concluded with a final elongation step of 10 min at 72 °C. After thermal cycling the PCR sample containing the first amplicon was diluted 1:50 in nuclease-free water. For the second PCR, 2 µL of the diluted PCR sample was combined with 0.125 µL dNTPS (40 mM, i.e., 10 mM each dNTP), and 2.5 µL 10X DreamTaq Buffer and 0.5 µL DreamTaq Polymerase (5 U/µL) (both Thermo Scientific, Waltham, MA, USA) were combined with 1.25 µL of each of the primers R16F2n (10 µM) and R16R2 (10 µM). To this mix, nuclease water was added to reach a final volume of 25 µL. The cycling conditions of the second PCR are the same as for the first PCR. An aliquot of 10 µL of every PCR sample was analyzed on a 1% agarose gel. Amplicons of the second PCR round were purified with QIAquick PCR Purification Kit (Qiagen, Hilden, Germany) and sent to an external sequencing service to perform Sanger sequencing (LGC Genomics, Berlin, Germany). Additional details on phytoplasma detection strategy and its limitations are provided in the Supplementary Materials.

Amplicon sequences were BLAST searched against the NCBI nucleotide database to confirm the presence of phytoplasma DNA in the analyzed sample. Sequences were then subjected to phytoplasma group identification using the iPhyClassifier online tool for phytoplasma classification and taxonomic assignment [37]. For subgroup identification, NCBI BLAST hits that were identical to the query and were assigned to an accession of sufficient sequence length for subsequent identification (i.e., at least 1245 nucleotides) were identified and used as the query for ‘*Candidatus* Phytoplasma’ species assignment and 16Sr classification based on RFLP patterns (subgroup determination) using the iPhyClassifier.

### 2.4. DAMA Protocol, Phylogenetic Triage and Risk Assessment

Insect data collected during the RII 2020 survey from 56 locations in the Bavarian region were organized in a final species checklist. Screening for the presence of phytoplasmas resulted in positive detection and, therefore, provided a valuable opportunity to evaluate the risk of potential phytoplasma outbreaks in the study region. The DAMA protocol was used as a workbench to evaluate the risk of outbreaks and to propose proactive actions to mitigate potential threats. The protocol provides general guidelines that were customized to be applied in the Bavarian region. The first component of DAMA is “Document: Make use of local knowledge in areas being sampled. Provide the following information: (i) what known pathogens occur in a place, (ii) where else do they occur, (iii) what are their reservoirs, (iv) what is their prevalence/distribution in populations of hosts, and (v) what environmental factors enhance their survival, and where do those conditions occur?” [2]. We evaluated the data collected during the RII 2020 collection and the latent phytoplasma infections detected during this biological sampling campaign. Moreover, different databases and checklists of species of plants, phytoplasmas and hemipteran insects were used to assess the risk of phytoplasma outbreaks in the study region. For plants, a vascular plants checklist for Bavaria is available from the “Botanischer Informationsknoten Bayern (BIB)” database [38]. We retrieved the list of plants recorded in Bavaria from more than 100 years ago up until the present day. According to BIB, 4000 vascular plant species have been recorded in Bavaria, including the three most economically important crops for the Bavarian region [39]: wheat (*Triticum aestivum*, Poaceae), barley (*Hordeum vulgare*, Poaceae) and maize, (*Zea mays*, Poaceae). A checklist of leafhoppers and planthoppers of Germany has been published previously [40,41,42], and the species were characterized based on their habitat and plant associations. The list of phytoplasmas was retrieved from the Hemiptera–Plant–Phytoplasma (HPP) interaction database [43], and all phytoplasma species and groups recorded in Germany (included in nine phylogenetic phytoplasma groups) were selected and further recent additions were also considered. After the evaluation of potential host reservoirs (insects and plants), we evaluated the environmental interfaces. The interfaces are boundary zones, of varying extents, that are the nexus for pathogen exchange across managed (anthropogenic) and wildland habitats. The evaluation of the interfaces was carried out for the macro-areas associated with presence of phytoplasmas after the screening of samples from the RII 2020 survey. The open-source geographic information system licensed under the GNU General Public License (QGIS v. 3.10) [44] was used to record the proportion of hedgerows and woodland remnants surrounding the grassland and crop fields.

**Figure 2:**
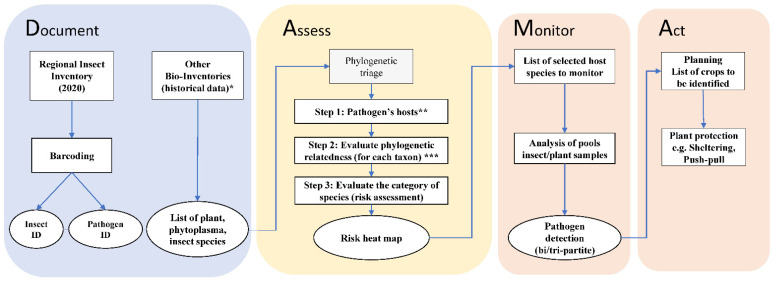
Flowchart of the DAMA (Document, Assess, Monitor, Act) protocol [6] applied for the Bavarian region, South Germany. Document: during a regional insect collection, micro-invertebrate samples were collected in 2020; this information was complemented using data from existing bio-inventories and database (*: [38,40,41,42,43]). Samples were processed using high-throughput molecular analysis (metabarcoding, for insect identification—ID) and sanger sequencing (for pathogenic bacteria identification). Assess: a three-step phylogenetic triage was applied for species risk assessment by using an existing global database (** Hemiptera-Phytoplasma-Plant Database [45]) and phylogenetic time trees (*** phylogenetic relatedness evaluated from Cao et al. [46] for leafhoppers; Kumar et al. [47] for plants; Cao et al. [48] for phytoplasmas). Monitor: host species selected from the Assess component are suggested for regional monitoring programs. Act: actions to plan to prevent emerging phytoplasmas disease and outbreaks. Boxes represent data and methods, ovals are interim and final results.

The second component of DAMA is “Assess: determine the suite of microbes of special concern that should be monitored closely. This is a two-part process. The first step is phylogenetic triage: place each discovered microbe in a phylogenetic context and ask if it is a known pathogen?” [2]. Based on the data collected during the documenting component, we performed a phylogenetic triage using the HPP database. In the database, the taxa are organized hierarchically, and the triage was customized in three steps: (1) selection of all plants, competent and potential vectors (hosts) of the target pathogen; (2) evaluation of the phylogenetic relatedness using previously published phylogenies; (3) evaluation of groups of host–pathogen associations with different categories of risk. The results of the phylogenetic triage were summarized in a risk heat map depicting the likelihood that the tripartite association (vector–phytoplasma–plant) will occur and its impact in the region under study (Figure 2). The last two components of DAMA are “Monitor: regularly re sample potential or known pathogens of interest in areas where they have been discovered and search for them in areas predicted to be suitable for them (vulnerable areas, such as areas of new introduction). Re-asses […] the pathogen population being monitored” and “Act: those responsible for disease-related food and public health security need to formulate action plans”. In this study, we provide actionable information for monitoring (Monitor) and discuss possible implementation in the field contributing to prediction and mitigation (Act). In Figure 2, an overview of the DAMA protocol customized for this specific study focusing on the Bavarian region is provided.

## 3. Results

### 3.1. Metabarcoding Insect Identification

Combining the blast results from the BOLD, NCBI and RDP classifiers, a total of 898 insect OTUs from metabarcoding were identified from a total of 228 micro fraction bulk samples (76 sampling sites × 3 periods of collection) collected in the four macro-areas. We selected Hemipetera Auchenorrhyncha, which include known hosts of phytoplasmas. A total of 136 consensus hemipteran OTUs were assigned to 34 genera and five families (Aphrophoridae, Cicadellidae, Cixiidae, Delphacidae and Membracidae) (Table S1). A total of 54 OTUs were identified to the family level (~40%), 82 OTUs to the genus level (~60%) and 36 to the species level (~26%). For application of the DAMA protocol (see Section 3.3 Phytoplasma-host associations and phylogenetic triage) we evaluated 82 OTUs identified at genus level, taxa identified at family level are poorly informative with respect to their status as vectors of phytoplasmas.

**Figure 3:**
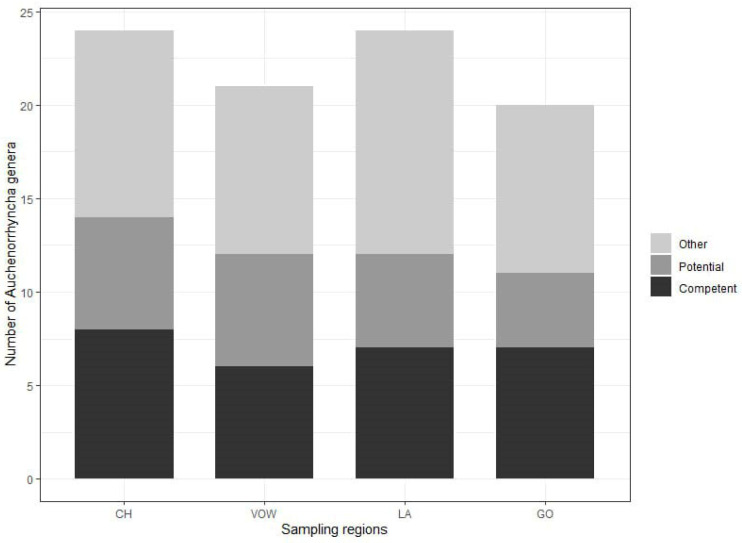
Number of identified Auchenorrhyncha genera for each sampling region obtained with Metabarcoding analysis of 228 insect samples. In light gray, the proportion of genera including at least one species for which no information about phytoplasma vector status is available. In dark gray, the proportion of genera including at least one potential vector of phytoplasmas. In black the proportion of genera including at least one competent vector of phytoplasma. On the x-axis, the acronyms for the sampling regions are as follows: CH, Chiemgau; VOW, Upper Palatine Forest; LA, Kelheim—Laaber and GO, Dachau—Glonn.

The minimum numbers of Auchenorrhyncha genera were 24 and 21, for the two grassland macro-areas, CH and VOW, respectively, and 24 and 20 for the two agricultural macro-areas, LA and GO, respectively (Figure 3, Table S1). The highest number of genera comprising known competent hemipteran vectors of phytoplasmas from the literature [45] was recorded in the grassland macro-area CH (eight genera: *Anoscopus*, *Cicadula*, *Hebata*, *Macropsis*, *Macrosteles*, *Oncopsis*, *Orientus*, *Psammotettix*). A further six genera comprising known potential vectors (*Aphrophora, Cicadella*, *Cixius*, *Eupteryx*, *Laodelphax, Philaenus*). In VOW macro-areas, a total number of 12 genera comprising known competent and potential vectors was recorded. In the GO agricultural macro-region seven genera (*Athysanus*, *Hebata*, *Euscelis*, *Macropsis*, *Macrosteles*, *Orientus*, *Psammotettix*) were recorded comprising competent vectors and four potential vectors (*Cicadella*, *Cixius*, *Eupteryx*, *Laodelphax*). In the second agricultural area (LA), seven genera comprising competent (*Aphrodes*, *Cicadula*, *Hebata*, *Macropsis*, *Macrosteles*, *Oncopsis*, *Psammotettix*) and five potential vectors (*Aphrophora*, *Cicadella*, *Cixius*, *Eupteryx*, *Laodelphax*) were recorded (Figure 3).

### 3.2. Detection of Phytoplasmas in the Study Region

Phytoplasma-specific DNA was detected in two out of the 152 malaise-trap samples (collected from 76 sampling sites during two periods of collection) (Table S2). The two sequences covering the nearly full-length *16Sr* gene (1155 bp and 1135 bp, respectively) were submitted to the first iPhyClassifier database for ‘*Candidatus* (*Ca*.) Phytoplasma (P.)’ species assignment and then to the second database for group and subgroup classification. Both sequences were classified as ‘*Ca*. P. asteris’-related strains sharing 99.65% and 99.56% sequence similarity with the reference strain (GenBank accession: M30790.1), respectively. These two phytoplasma strains belong to the aster yellow (16SrI) group and were further classified in two phytoplasma subgroups: 16SrI-L (1209-F5-W, from region LA, sampled in September) and 16SrI-B (1396-F8-M, from region GO, sampled in September). The 16SrI-L and 16SrI-B strains share 100% similarity with GU223209.1 and M30790.1 reference strains, respectively (Figure S1).

### 3.3. Phytoplasma–Host Associations and Phylogenetic Triage

The discovery of two ‘*Ca*. P. asteris’-related strains in the Bavarian region provided an extraordinary opportunity to apply the DAMA protocol using the data from archives, biorepositories and databases available in the literature, including the data from the recent RII 2020 survey. An initial literature search revealed no previous reports of outbreaks of aster yellow diseases associated with ‘*Ca*. P. asteris’ in the Bavarian region. For the first component of DAMA (*Document* Figure 2) we answered the following questions:

(i) What known pathogens occur in a place (Bavarian region)? Five phytoplasma records were available in the literature for Bavaria: ‘*Ca*. P. ulmi’ [49], which were recorded near the GO region, ‘*Ca*. P. mali’ [50], ‘*Ca*. P. prunorum’, ‘*Ca*. P. rubi’, ‘*Ca*. P. solani’ [51]. Only the last one is closely related to the ‘*Ca*. P. asteris’ group.

(ii) Where else do they occur? We searched other records for Germany, and a total of nine phytoplasma 16Sr groups had previously been recorded: ‘*Ca*. P. asteris’ and related strains in the subgroups 16SrI-B, -C and -M subgroups [52,53,54,55,56,57]), 16SrIII phytoplasma group [54], ‘*Ca*. P. ulmi’, alder yellow phytoplasma (16SrV-C), ‘*Ca*. P. rubi’ [58,59,60,61,62], ‘*Ca*. P. mali’, ‘*Ca*. P. prunorum’, ‘*Ca*. P. pyri’, 16SrX-E [54,63,64,65,66,67]; 16SrXI [54,67]; 16SrXII [68,69,70]; 16SrXIV [67]; 16SrXX [71] and 16SrXXI [72] phytoplasma groups.

(iii) What are their reservoirs? Altogether, the pathogens listed above, belonging to nine different phytoplasma phylogenetic groups, were recorded on at least 33 plant species, including *Alnus glutinosa*, *A. hirsuta*, *A. subcordata*, *A. rugosa*, *A. tenuifolia*, *Aquilegia alpina*, *Bunias orientalis*, *Callistephus chinensis*, *Calystegia sepium*, *Cardaria draba*, *Cirsium arvense*, *Convolvulus arvensis*, *Cuscuta odorata*, *Cyclamen persicum*, *Daucus carota*, *Delphinium hybrid*, *Malus domestica*, *Pinus sylvestris*, *Plantago* sp., *Populus alba*, *P. nigra*, *P. tremula*, *Primula* sp., *Prunus* spp., *Rhamnus catharticus*, *R. frangula*, *Rubus idaeus*, *Solanum nigrum*, *Stellaria media*, *Trifolium repens*, *Urtica dioica*, *Vaccinium myrtillus*, and *Vitis vinifera*, and 14 insect vectors (*Anoscopus albifrons*, *Aphrodes bicinctus*, *Cacopsylla picta*, *C. pruni*, *C. pyri*, *C. pyricola*, *Fieberiella florii*, *Hyalesthes obsoletus*, *Idiocerus stigmaticalis*, *Macropsis fuscula*, *Macrosteles laevis*, *Oncopsis alni*, *Psammotettix cephalotes*, *Psylla* sp.) [43].

(iv) What is their prevalence/distribution in populations of hosts? Specific patho-systems (e.g., alder yellow phytoplasma-related strains, *Oncopsis alni* and *Alnus* spp.) are locally distributed (i.e., in the Upper Palatinate Forest) [60] and may show high prevalence of infection. Another well-known patho-system (e.g., ‘*Ca*. P. solani’, *Hyalestes obsoletus* and *Convolvulus arvensis* and *Urtica dioica*) in Germany presents endemic characteristics, with several outbreaks re-emerging over time in different parts of Germany [73,74]. In the present study we focus on the Bavarian region and we selected the phytoplasma ‘*Ca*. P. asteris’ and other related strains which were also reported in other regions in Germany with sporadic outbreaks (Kube M., pers. com.). For this group, the known hosts plants are: *Cuscuta odorata*, *Populus alba*, *Populus nigra*, *Populus tremula* (woody area), *Callistephus chinensis* (ornamental), *Vitis vinifera, Daucus carota* (crops) [43].

(v) What environmental factors enhance their survival, and where do those conditions occur? For phytoplasma strains to survive and spread, an established association among plants and their vectors must persist across a certain area. Considering ‘*Ca*. P. asteris’-related strains, the dynamic of the patho-system may vary if the plant is annual or perennial and/or if the vectors have a narrow or a broad range of host plants. The Bavarian region is characterized by two main landscapes: crop lands and grasslands. The crop lands are dominated by annual crops such as winter wheat (*Triticum aestivum*), winter rye (*Secale cereale*), winter and summer barley (*Hordeum vulgare*), rape (*Brassica napus*), winter triticale (*Triticale*), sugar beet (*Beta vulgaris*), maize (*Zea mays*), legumes. Considering the field-landscape interface of the 20 crop field locations in GO and LA sampled during the RII 2020 survey, at least one big woody patch was present in the vicinity of about half of the fields, and several hedgerows were recorded in almost all the fields (Table S3). One hedgerow and one big woody area were adjacent to the sites where the two phytoplasmas strains were detected, F5-W (LA) and F8-M (GO), respectively. The interfaces between the crop fields and hedgerows or woody areas may represent a suitable environment to support the survival and spread of phytoplasmas into the crop fields. Potential for disease outbreaks will depend on the susceptibility of plant hosts and on the vagility and host preferences of the insect vectors, these factors have to be considered for the early detection of phytoplasmas.

For the second component of DAMA (*Assess* Figure 2), we applied the three-step phylogenetic triage focusing on the two ‘*Ca*. P. asteris’-related strains recorded in the Bavarian region and its potential hosts. The aim of the triage is to use information on evolutionary relationships to assign levels of risk to known or potentially new host–pathogen associations that may result in disease outbreaks.

(1) First step: Pathogen’s known host range (*Assess*, Figure 2). Based on the HPP database, 22 hemipteran species were recorded as competent vectors of ‘*Ca.* P. asteris’ related strains. Insect hosts are unknown for the 16SrI-L phytoplasma subgroup. We discarded nine species limited to biogeographic regions outside the Palearctic. Among the remaining 13 species (Table 1, species with a single asterisk), three (*Scaphoideus titanus*, *Osbornellus horvathi* and *Adarrus taurus*) had not yet been recorded for Germany, one (*Hardya tenuis*) was not recorded in the Bavarian region, two (*Macrosteles quadripunctulatus* and *Neoaliturus fenestratus*) were uncommon in the Bavarian region and seven (*Hebata decipiens*, *Euscelis incisa*, *E. lineolata*, *Euscelidius variegatus*, *Macrosteles laevis*, *Psammotettix alienus*, *Athysanus argentarius*) were widespread. Excluding *S. titanus*, *O. horvathi*, *A. taurus*, and *H. tenuis*, a total of nine species (representing six species and three species groups in *Euscelis*, *Macrosteles* and *Psammotettix*) were selected for the next step of the triage (phylogenetic tree).

Phylogenetic conservatism in traits related to resource use (widespread host-based resources, in contrast to specific host species) allows rapid host colonization through ecological fitting [21,75,76,77]. Previous studies on hemipteran vector–phytoplasma interaction showed that phylogenetic conservatism is expressed at least at the level of the tribe [43]. To take phylogenetic conservatism into account, we selected five species recorded as competent vectors of other 16Sr phytoplasma groups (16SrIII, 16SrV, and 16SrXII) and that are closely related (same tribe) to the competent vectors of ‘*Ca.* P. asteris’ strains (Table 1, species with double asterisk). Among them, two species (*Anoplotettix fuscovenosus* and *Orientus ishidae*) were selected for the next step of triage as an outgroup for two reasons: they are widespread in Germany (including the Bavarian region), and they are also associated with more distantly related phytoplasma groups.

According to the HPP database, 161 plant species, representing 59 families, were recorded as hosts of ‘*Ca.* P. asteris’ strains. Almost all the plant species recorded are crops, and for this reason, the vast majority are widely distributed, and some of them are not of Palearctic origin but are naturalized [43]. A total of 25 species were discarded because they were strictly distributed in other biogeographic areas and not recorded in the Palearctic region. The 136 remaining species representing 54 families were evaluated and 24 species representing 16 families previously recorded as hosts of ‘*Ca.* P. asteris’ were selected for the second step of the triage [43] because they are commonly found in the Bavarian region. Among them, 12 species (*Brassica* sp., *Corylus avellana*, *Daucus carota*, *Malus domestica*, *Papaver rhoeas*, *Plantago* sp., *Salix* sp., *Solanum tuberosum*, *Taraxacum* sp., *Trifolium* sp., *Triticum aestivum*, *Zea mays*) belonging to 11 families were recorded with high occurrence in the sampled areas in the Bavarian region.

**Figure 4:**
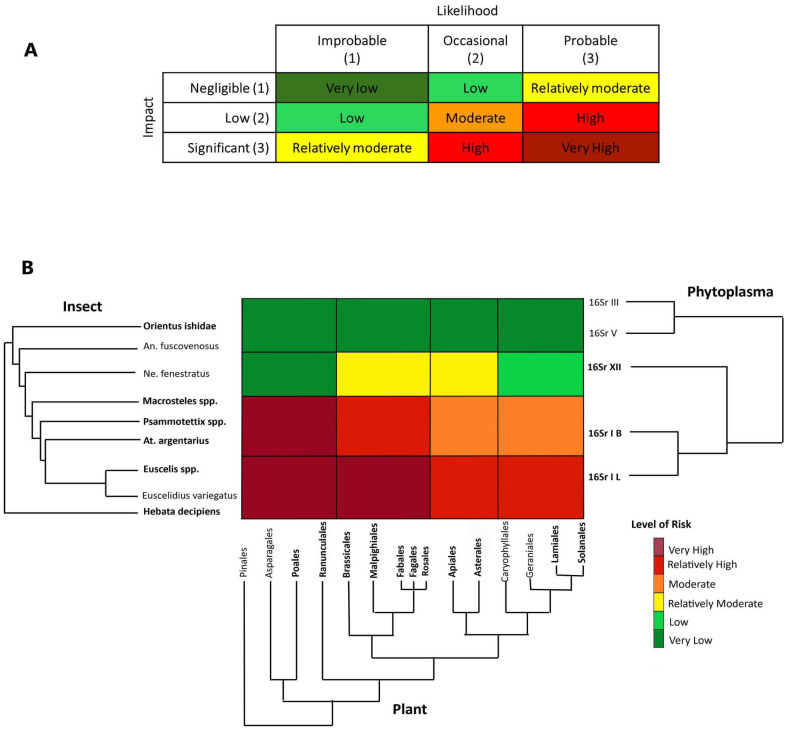
Risk heat map based on six levels of risk from very high to very low (**A**). Evolutionary-based risk heatmap derived from the results of the three step phylogenetic triage (**B**) representing different outcomes of simultaneous presence in the study region and in the sampled locations and the historical records of bipartite associations (phytoplasma–insect vector, phytoplasma–plant, plant–insect vector) or tripartite associations. Taxa highlighted in bold were recorded during the RII 2020 survey in the Bavarian region. Abbreviations: An. fuscovenosus = *Anoplotettix fuscovenosus*, Ne. fenestratus = *Neoaliturus fenestratus*, At. argentarius = *Athysanus argentarius*.

(2) Second step: Evaluate phylogenetic relatedness of pathogen hosts. The phylogenetic reconstruction for the selected 11 hemipterans is based on the highly resolved backbone phylogeny of Deltocephalinae available in [46]. For the final phylogeny we selected nine genera (*Anoplotettix*, *Athysanus*, *Euscelis*, *Euscelidius*, *Hebata*, *Macrosteles*, *Neoaliturus*, *Orientus*, *Psammotettix*) and we considered *Euscelis*, *Macrosteles* and *Psammotettix* as a group because they are relatively closely related, share the same ecological preferences, and some of them are morphologically cryptic (Figure 4, phylogeny on the left). Three species and three species groups (*H. decipiens*, *Euscelis* spp., *A. argentarius*, *Psammotettix* spp., *Macrosteles* spp., *O. ishidae*) (Figure 4, in bold) were recorded in the same locations where the ‘*Ca*. P. asteris’-related strains were detected during the RII 2020 survey. In the phylogenetic tree the monophyletic deltocephaline leafhopper group of Athysanini + Macrostelini + Paralimnini + Opsiini (*Euscelis* sp., *A. argentarius*, *Psammotettix* sp., *Macrosteles* sp., *O. ishidae*) is distantly related to the typhlocybine tribe Empoascini (*H. decipiens*) which hosts the ‘*Ca*. P. asteris’ pathogen. Thus, we can infer that the trait(s) explaining the host–pathogen interactions with both of these leafhopper groups is/are plesiomorphic.

For plant phylogeny reconstruction, we used the time tree reconstruction from Kumar et al. [47] using a phylogenetic tree showing relationships among orders of angiosperm species recorded as ‘*Ca*. P. asteris’ hosts (Figure 4, phylogenetic tree on the bottom). The 25 plant species selected represent 16 families and 15 orders, among them 11 species representing 11 families and 11 orders were recorded with high occurrence in the sites sampled in the Bavarian region (Figure 4, plant orders in bold).

For the phylogenetic reconstruction of phytoplasmas, we selected the strains recorded for the first time for the Bavarian region during the RII 2020 survey, belonging to both ‘*Ca*. P. asteris’ and three phytoplasma groups (16Sr-XII, -V and -III) from the two major clades detected in previous phylogenetic reconstructions [48] that were recorded in Germany.

The phylogenies of the two groups of hosts (vectors and plants) were plotted into the phylogeny of the phytoplasmas to evaluate historical events of geographical and host colonization of phytoplasmas. The ancestor of the 16SrI + 16SrXII phytoplasma clade arose approximately 115 million years ago (Mya), roughly coinciding with the divergence of major lineages within Typhlocybinae, including Empoascini (~99 Mya, [78]) and the large grass lineage, Poales (~107 May, [47]). All the major groups of forbs (i.e., Ranunculales, Brassicales and Asterales) diverged between 128 and 85 Mya ago. The crown clade of 16SrI + 16SrXII was dated 145 Mya and the subclades 16SrXII and 16SrI began to diversify during the Paleogene, about 63 and 35 Mya [48] roughly coinciding with the divergence of Opsiini, Paralimnini, Macrostelini and Athysanini (including the species group in Table 1), 69, 52, 53 and 40 Mya, respectively [46].

(3) Third step: Assign species and associations to risk categories. The risk assessment was performed using a heat map that depicts the risk for phytoplasma outbreaks in the region under study across three axes corresponding to phylogenies for hemipteran insects, plants and phytoplasmas. Briefly, the axes roughly correspond to distances along branches of the phylogenies of the three groups of potentially interacting organisms and each cell’s color indicates the value of the risk (likelihood and impact) in the corresponding cell range based on the inferred potential for interaction between pathogens, potential vectors, and host plants. Each cell was interpreted as a tri-partite co-phylogeny achieved by overlapping the three phylogenies, evaluating the current and ancient associations, and assigning the final-colored risk category (from very low to very high).

The risk of potential aster yellow disease outbreaks associated with ‘*Ca*. P. asteris’ and closely related phytoplasma strains in the Bavarian region was evaluated by considering historical ecology (e.g., [79]) and documented associations that represent the likelihood of particular associations occurring, tripartite co-occurrence of the organisms in the Bavarian region and potential economic impact on the major crops cultivated in that region. The risk is given by *likelihood* × *impact* (Figure 4A). From an historical ecology perspective, the clade of 16SrI + 16SrXII phytoplasmas is associated with the paraphyletic group (Empoascini + Opsiini + Macrostelini + Paralimnini + Athysanini). The radiation of the 16SrI phytoplasma group shortly following the emergence of the lineage of grass-specialist leafhoppers (Macrostelini + Paralimnini + Athysanini) suggests that a contraction of the pathogen’s host range occurred, mediated by grass specialization in this lineage of potential vectors. At a short temporal scale, the observed current preferences of certain strains of the 16SrXII phytoplasma group for Opsiini leafhoppers and herbaceous dicots (forbs) was recently demonstrated by Mitrović and colleagues [80]. Thus, the likelihood of association with ‘*Ca*. P. asteris’ strains and closely related phytoplasmas happening is probable (3) for *H. decipiens*, *E. variegatus*, *Euscelis* spp., *Athysanus argentarius*, *Psammotettix* spp., and *Macrosteles* spp. (representing Empoascini, Athysanini, Paralimnini and Macrostelini, respectively), occasional (2) for the opsiine species *N. fenestratus* and improbable (1) for the more distantly related deltocephalines *A. fuscovenosus* and *O. ishidae*. By taking the perspective of pathogens in the aster yellows group (16SrI) and considering the potential impact of aster yellow disease in this region, the risk of outbreak is assigned as follows:

For the paraphyletic group including *H. decipiens* and *Euscelis* spp. we assigned a risk from very high to relatively high (Figure 4). In particular, *H. decipiens* was recorded as a pest on barley [81], a crop present in the study region, and was recorded as a competent vector of ‘*Ca*. P. asteris’ phytoplasma [82]. The genus *Euscelis* includes a group of species known to be associated with grasses and forbs (legumes in the order Fabales), with one species, *E. incisa*, that has been demonstrated to be able to acquire and inoculate several strains in the phytoplasma clade (16SrI + 16SrXII) [83]. Because both species were recorded in almost all crop fields during the RII 2020 survey, including the sites where we detected two ‘*Ca*. P. asteris’-related strains, we assigned a likelihood of association with insects and potential susceptible crops as probable, and the economic impact on the major crops significant (overall risk, very high; Figure 4B). The plant clade including Apiales, Asterales, Caryophyllales, Geraniales, Lamiales and Solanales, may host *H. decipiens* and *Euscelis* spp., but these orders do not include major crops in the studied region, and although the association with the plant hosts is probable, the impact was rated as low, while the overall risk is relatively high (Figure 4B).

For the paraphyletic group including *A*. *argentarius* (Athysanini), *Psammotettix* spp. (Paraliminini) and *Macrosteles* spp. (Macrostelini), we assigned a risk from very high to moderate. All the species in this group were recorded in the study region with high incidence (notably *Psammotettix* spp. and *Macrosteles* spp.) and are known to be associated with various grasses including the major crops in the study region. As a consequence, the association with Poales is very probable, with a potential significant impact in the studied region (the overall risk, Very High Figure 4B). *A. argentarius* is considered to play an important role in maintaining aster yellow disease in reservoir plants in the interfaces (ditches, ecotones, and abandoned fields) of canola (Brassicales) fields [84] in Canada. The species has also occasionally been found in clover (Fabales) fields in Europe [85]. The aster leafhopper, *Macrosteles quadrilineatus* is also known to be the major vector in *Brassica* crops in the Nearctic region [86,87], whereas the record of European species in the genus *Macrosteles* as putative vectors of aster yellows in rape crop is quite old [88] and has not been confirmed recently. For these reasons, in the Bavarian region, we consider the association of this group of species with the plant clade including Brassicales to be occasional, but the impact significant, with the resulting risk of an outbreak in rape or legumes being relatively high. The association with plants in other orders may be occasional and the impact low, with a resulting moderate risk (Figure 4B).

*Neoaliturus fenestratus* was reported as a potential competent vector of aster yellows using manipulative transmission trials (feeding medium) [89], and recent evidence has shown a major role as a vector of stolbur phytoplasma (‘*Ca*. P. solani’) [80]; thus, we assigned a risk from relatively moderate to very low (Figure 4). Because the species was not detected during the RII 2020 survey, and it is uncommon in the Bavarian region, we considered the likelihood of association with plants and phytoplasmas in the region improbable (even if possible due to migration and introduction), whereas the impact of a potential outbreak is negligible on grasses, low on the clade of forbs including Solanales, and significant on forbs, such as Brassicales and Asterales [90,91]. The paraphyletic group including *O. ishidae* and *A. fuscovenosus* is considered here as an outgroup from the 16SrI pathogen’s perspective, as they are strictly associated with distantly related groups of pathogens (16SrIII and 16SrV); thus, we assigned a very low risk with a likelihood of association improbable and impact negligible.

According to the phylogenetic risk heat map, at least seven species (*Athysanus argentarius*, *Euscelidius variegatus*, *Euscelis* spp., *Macrosteles* spp., *Hebata decipiens*, *Psammotettix* spp., and *Neoaliturus fenestratus*) need to be considered for the last two components of DAMA (“Monitor” and “Act”) and the implications are discussed below.

## 4. Discussion

### 4.1. Why Do We Need a Proactive Approach for Phytoplasma Diseases?

Over the past century, the prevalent idea that emerging infectious plant diseases (EIPD) are isolated and rare events led stakeholders to focus almost exclusively on crisis management [3]. Only after diseases emerge or re-emerge are massive resources allocated to cope with pathogens that have become highly prevalent across an area and have made themselves known by causing significant economic losses. Surveillance and preventive actions have been suggested to cope with EIPD [92]; however, a proactive approach is more powerful, because it is better-informed for coping with emerging disease crises before outbreaks occur, with greater potential to save time and resources [93]. Very few efforts have been undertaken to reveal the much greater threat posed by plant pathogens “waiting behind the scenes” during times in which no outbreaks are being recorded in the area under study [94].

Therefore, an evolutionary perspective to characterize the potential for plant pathogens to invade new habitats and hosts provides a predictive power for EIPD and is urgently required. This perspective has recently been suggested and is summarized in the theoretical integrative framework of the Stockholm Paradigm [9,76] as well as its policy extension, the DAMA protocol [95,96]. In brief, the framework is founded on gathering information from different sources (see [7] for an overview) and on the inclusion of an eco-evolutionary analytical approach. Substantial actions can be taken to predict or mitigate the impacts of emerging diseases that may be adapted from global to local level. The potential for EIPD relies on the evolutionary history of pathogens and on ecological perturbations, such as climate change, habitat disruption and biological invasions. 

Phytoplasma-associated diseases pose a severe threat to different important agricultural crops [15] and continue to emerge at a steady pace [97,98]. However, despite the highly advanced currently deployed methods, knowledge of phytoplasma global diversity and distribution is still highly incomplete, even in relatively well-studied areas such as Europe [18]. As postulated by Audy [99] for animal pathogens, observing the absence of the phytoplasma disease in certain areas, we could assume that the distribution of the pathogen is larger than the disease that it causes. Even if a phytoplasma disease is absent, it does not mean the associated pathogen is. Earlier studies have reported the importance of phytoplasma source of inoculum in non-crop plants (reservoirs) (e.g., [100]). Potential geographic expansion of compatible insect vectors may allow rapid invasions from infected reservoirs in not cultivated areas to susceptible crops in agroecosystems, as predicted for zoonotic diseases [6].

### 4.2. Applying the DAMA Protocol to Reduce the Costs of Potential EIPD

In this study, we applied the DAMA protocol to assess the risk of potential emerging vector-borne phytoplasma disease. This protocol was successfully applied to vector-borne zoonotic pathogens (e.g., [101]) and other animal diseases, and it was suggested recently for phytoplasma-associated diseases [2,102], but this is the first time the protocol has been applied to a case study at the regional level. Notably, we used data already on hand (such as archives and databases; Document in DAMA) and recent findings of silent phytoplasma infections in Bavaria, South Germany. Silent infections are considered to occur in reservoir host plants or in agriculturally relevant crops in an asymptomatic manner. For this study, we used a PCR-based approach to detect phytoplasma presence in mass-trapped and pooled insect samples collected in grasslands and croplands. In previous studies it has been suggested that screening hemipteran vectors may be a more efficient way of tracking silent infections, rather than screening wild plants [17,103]. Although we are aware that merely ingesting phloem sap during feeding or probing is not evidence for vector competence, we used phytoplasma detection in this study as evidence for the pathogen circulating in the environment in a still-unknown repertoire of hosts in Bavaria. In our study, we detected two ‘*Ca*. P. asteris’-related strains, belonging to 16SrI-B and 16SrI-L phytoplasma subgroups, the last one never detected before in the Bavarian region. The detection anticipates potential phytoplasma outbreaks and allowed us to use the DAMA protocol to evaluate potential insect and plant hosts (including crops). Using a phylogenetic triage (Assess, in DAMA), we selected at least seven leafhopper species, i.e., *Hebata decipiens*, *Euscelius variegatus*, *Euscelis* spp., *Macrosteles* spp., *Psammotettix* spp., *Athysanus argentarius*, and *Neoaliturus fenestratus*, that may serve as vectors of ‘*Ca*. P. asteris’-related strains and pose from “relatively moderate” to “very high” risk for phytoplasma outbreaks in economically important crops in Bavaria. Some key elements of the DAMA approach are the use of sophisticated archival repositories, bioinformatics, next-gen analyses, and phylogenetic triage to assess the risk of potential associations. Another important point is that the assessment considers habitat interfaces vegetated with non-cultivated plants and inhabited by phloem-feeding insects that may serve as reservoirs. More importantly, narrowing the search for potential vectors and reservoirs, the protocol makes it possible to save time and resources, because the screening of the entire biodiversity of hosts or pathogens in croplands and in the interfaces with other habitats is not sustainable and cost effective. We suggest that stakeholders in collaboration with agronomic research institutes in Bavaria launch specific monitoring programs to validate in the field the potential prevalence of infection in the population of the selected insects at the interfaces with fields of susceptible crops where the risk of outbreaks is higher. The DAMA protocol embodies a modeling platform (such as the Individual-Based Model [104,105]) that explores fitness profiles of pathogens and the probability of an individual in a population to disperse and successfully colonize a novel host. A targeted monitoring plan may generate data for the modeling platform to narrow our focus to particular pathogens and vectors in specific habitat interfaces and plant hosts.

### 4.3. Economic Importance of Aster Yellow Phytoplasma in the Study Region

Although aster yellow phytoplasma (‘*Ca*. P. asteris’) is listed as a non-quarantine organism according to Regulation (EU) 2019/2072, which considers protective measures against pests of plants [106], it may still pose a potential threat, and we suggest specific monitoring actions at the regional level (i.e., Bavaria). Such monitoring plans should be defined to track latent infections long before they become problematic. Aster yellow disease was observed in wheat in Canada in the 1960s and included symptoms such as dwarfing, chlorotic blotching, chlorosis, necrosis and premature death [107], and it still causes severe yield losses in wheat production [108]. Recently, recrudescence of aster yellow disease was observed in Saskatchewan (Canada) (https://saskwheat.ca/news-articles/aster-yellows-feb-2020, accessed on 10 May 2023). ‘*Ca*. P. asteris’ is a pathogen of concern for vegetable farmers in the American Midwest, and a recent study defined *Macrosteles quadrilineatus* as playing a major role in maintaining high rate of infections in the crop fields [109]. Several growers cope with the disease through regular applications of pyrethroid insecticides with known adverse effects on non-target wildlife and humans [110,111]. Because of the specific biological behavior of this leafhopper vector, which migrates from central America to South Canada each spring, insecticide application cannot be the only approach in place but has to be supported by a proper monitoring plan.

Significant losses occurred in China due to wheat blue dwarf disease (associated with 16SrI-C phytoplasma subgroup), with an average total loss of 50 thousand tons each year during the epidemic. The vector *Psammotettix striatus* (a synonym of *Psammotettix alienus*) has been reported to be the only one transmitting the blue dwarf phytoplasma to wheat in China [112,113]. Moreover, a multilocus genetic approach provided evidence for this phytoplasma belonging to a different subclade comparing other 16SrI-C strains and, in this specific biogeographic region, showed distinctive association with plant hosts [112].

Aster yellow phytoplasma and related strains are widespread all over Europe, and they are associated with different diseases of vegetable and ornamental crops, but rarely with annual crops of high agricultural relevance. There have been recent reports of 16SrI-B and -L phytoplasma associated with rapeseed phyllody in France, Italy, Lithuania and Poland [114,115,116], and thus these strains pose a potential threat for rapeseed (*Brassica napus*) production in Germany, as well. Moreover, 16SrI-L phytoplasma has been detected on corn (*Zea mays*) in Poland [117] and 16SrI phytoplasma on sugar beets (*Beta vulgaris*) in Hungary [118]. 16SrI-B/L-related phytopathogenic phytoplasma strains have been detected in many different crop plants in Poland [119]. To the best of our knowledge, there have been no reports of ‘*Ca*. P. asteris’-associated diseases on major crops in Bavaria (i.e., corn, barley, sugar beet or rye). Applying the DAMA protocol in the Bavaria region, we suggest a proactive approach to prevent or mitigate potential aster yellow disease outbreaks. 

A similar study was carried out earlier in Serbia for other phytoplasmas listed as non-quarantine pests in Europe (stolbur phytoplasma, ‘*Ca.* P. solani’). Stolbur phytoplasma causes a range of diseases involving a plethora of natural reservoir plants, with the main vector being *Hyalestes obsoletus*, along with several other listed potential vectors. Mitrović et al. [120] provided a platform to assess the potential risks of re-emerging stolbur phytoplasma epidemics. To do so, the authors compiled an easy-to-use compendium listing all documented stolbur phytoplasma hosts (plants and insect vectors) and provided a scheme to policy officials and public decision making. The framework proposed by Mitrović and colleagues is closely akin to the DAMA protocol because endorse the importance of actionable tools for Documenting (compendium in Mitrović et al. [120] and existing databases and archives in DAMA) and Assessing (a risk heat chart to rank the likelihood of the risk in Mitrović et al. [120] and a co-phylogenetic risk heat map in DAMA). Because stolbur phytoplasma outbreaks may cause severe impacts in Europe on different crops such as grapevine, potato, and corn, the authors also suggested the use of the DAMA protocol to predict and cope with potential outbreaks. We believe that the DAMA protocol can be generalized to many other phytoplasma strains globally, while combining ‘boots on the ground’ contributions of citizen scientists, accumulated knowledge (such as biodiversity investigations see reference in this study), and tools already developed at regional level (such as the compendium and risk management scheme in [120]).

Using samples from a bioinventory study and a next-gen approach, we identified two strains of phytoplasmas in Bavaria; we applied the first two (Document and Assess) parts defined in the DAMA protocol and provided a list of insect species (potential vectors) suggested for the next step, i.e., Monitoring. This part has to be conceived and structured by stakeholders in Bavaria, and will provide the data necessary for reassessment, including specific knowledge of the real spatial distribution of the risk. The last step (Act) must be implemented through different initiatives, including but not limited to the use of actionable information for dissemination and public involvement as well as preventive actions following early detection.

## 5. Conclusions

Based on the results of the first two part of DAMA (Document, Assess, Monitor, Act) protocol and the risk assessment, we concluded that there is a considerable risk of outbreaks of 16SrI-associated diseases in economically important crops in Bavaria, i.e., wheat, barley, maize, rapeseed and sugar beet. Based on the phylogenetic triage, a list of at least seven insect species that may serve as primary (transmitting to crop of interest) or secondary (transmitting to non-crops but spreading phytoplasmas across the interfaces) vectors of aster yellow phytoplasmas and related strains was provided. We suggest the implementation of specific monitoring programs to be undertaken by the interested stakeholders. These programs should include diagnostic evaluation of phytoplasma presence using randomized samples at regular intervals. Monitoring strategies should include landscape interfaces, e.g., passive sampling techniques, such as yellow sticky traps or pan traps, could be placed at the transitional areas between crop fields and other non-cultivated green areas (forest, hedgerows, and other ecological compensation areas). This allows the early detection of insect vector and phytoplasma presence, especially before first symptoms occur in agriculturally relevant crops or in a state in which the disease is not yet widespread and it can still be contained. A proper investment in such proactive actions permits the detections of the pathogen in its silent stage, but requires some degree of cost. A reactive investment strategy accepts the risk of imminent or future outbreaks without accountability to avoid unnecessary expenditures.

## Figures and Tables

**Table 1:** List of Palearctic hemipteran species in the family Cicadellidae recorded as competent vectors of ‘*Ca.* P. asteris’ (*); other species from the same tribes were selected because they are competent vectors of other phytoplasmas (**). Historical records on habitat, host plant and distribution in the Bavarian region and Germany were collected from published literature and online databases [41]. NR: not recorded; W: widespread; Un: uncommon. The last column indicates the regions (VOW, Upper Palatine Forest; LA, Kelheim—Laaber; GO, Dachau—Glonn; CH, Chiemgau) where the species was identified using metabarcoding analysis during the Regional Insect Inventory 2020 (RII 2020). In bold the species (six species and three species groups) selected for the next step of the triage.

Subfamily	Tribe	Species	Habitat	Host Plant	Bavarian Region/Germany	RII 2020
Typhlocybinae	Empoascini	***Hebata decipiens*** *	ruderal areas, fields	weeds, shrubs, various crops	W/W	CH, VOW, GO, LA
Deltocephalinae	Athysanini	***Euscelidius variegatus*** *	ruderal areas	forbs	W/W	-
Deltocephalinae	Athysanini	***Euscelis incisa*** *	meadows, pastures	grasses and legumes	W/W	GO
Deltocephalinae	Athysanini	***Euscelis lineolate*** *	meadows, pastures	grasses and legumes	-/Un	GO
Deltocephalinae	Macrostelini	***Macrosteles quadripunctulatus*** *	sandy areas and viticultural region	grasses	Un/Un	CH, VOW, GO, LA
Deltocephalinae	Macrostelini	***Macrosteles laevis*** *	ruderal areas	grasses and forbs	W/W	CH, VOW, GO, LA
Deltocephalinae	Opsiini	***Neoaliturus fenestratus*** *	dry meadows, disturbed areas, fields	forbs	Un/W	-
Deltocephalinae	Paralimnini	***Psammotettix alienus*** *	meadows, disturbed areas, fields	grasses	W/W	CH, GO, LA
Deltocephalinae	Athysanini	***Athysanus argentarius*** *	meadows, fields, open forests	grasses	W/W	GO
Deltocephalinae	Athysanini	*Hardya tenuis* *	forest interfaces, grassland	trees and grasses	-/Un	-
Deltocephalinae	Paralimnini	*Adarrus taurus* *	-	-	NR/NR	-
Deltocephalinae	Scaphoideini	*Osbornellus horvathi* *	disturbed areas, fields	forbs and various shrubs	NR/NR	-
Deltocephalinae	Scaphoideini	*Scaphoideus titanus* *	disturbed areas, fields	*Vitis* spp.	NR/NR	-
Deltocephalinae	Scaphoideini	***Anoplotettix fuscovenosus*** **	forest margins, fields	forbs and various shrubs	W/W	-
Deltocephalinae	Athysanini	***Orientus ishidae*** **	forest margins, fields	woody and deciduous trees	W/W	CH, GO
Typhlocybinae	Erythroneurini	*Zyginidia scutellaris* **	ruderal, disturbed grassland, maize, winter cereals	grasses	Un/Un	-
Deltocephalinae	Athysanini	*Laylatina inexpectata* **	-	-	NR/NR	-
Deltocephalinae	Athysanini	*Thamnotettix dilutior* **	forests	trees and grasses	NR/W	-

## Data Availability

The data presented in this study are available in Supplementary Materials and on request from the corresponding author.

## Supplementary Materials

The following supporting information can be downloaded at: https://www.mdpi.com/article/10.3390/biology12050732/s1, Figure S1: Sequence alignment of a 16S rRNA gene stretch amplified in samples 9-A9 and 9-C12; Table S1: Taxonomic composition of the Hemiptera Auchenorrhyncha assemblages in 76 insect samples (40 in crop fields from Labber (LA) and Gloon (Go) macroareas and 36 from grasslands from Upper Palatine Forest (VOW) and Chiemgau (CH) macroareas), from 56 locations in the Bavarian region and collected during 3 sampling periods (1, June; 2, July; 3: September). COI family level or lower metabarcoding data resulting from the consensus taxonomy of three databases (BOLD, NCBI and RDP classifier); Table S2: List of samples analyzed for the presence of phytoplasmas and other bacterial pathogens in the class of Mollicutes and Gammaproteobacteria; Table S3: Number of landscape features in the surrounding locations investigated in regions Gloon (GO) and Labber (LA).
