# Supplementary material for: Phylogenetic Triage and Risk Assessment: How to Predict Emerging Phytoplasma Diseases
Source: Biology (Basel). 2023 May 17;12(5):732. doi: 10.3390/biology12050732 (PMC10215655; doi:10.3390/biology12050732)
Supplement: Supplementary file 1 [file biology-12-00732-s001.zip › Supplementary Material_revised.pdf]

SUPPLEMENTARY MATERIALS

Rationale for choosing endpoint nested PCR and Sanger sequencing.
A preliminary experiment was carried out to compare the sensitivity of two approaches to detect the presence of phytoplasmas on insect samples, TaqMan and nested-PCR. We used a series of tenfold dilutions (from 1:10 to 1:100.000) of the same sample. The nested-PCR resulted in a band of approximately 1.2 kbp in each dilution corresponding to the F2nR2 regionof the 16S rRNA gene targeted with the specific primer pairs (second PCR of the nested PCR) described in the Material and Methods section of the present paper. The qPCR-approach (TaqMan) (using phytoplasma species specific primers) resulted in Cq values ranging from 32.72 to 33.05 for a dilution of 1:10.000, 29.72 to 29.82 for 1:1000, 26.35 to 26.4 for 1:100, and 22.98 to 23.26 for 1:10. No Cq signal was detected with a template dilution of 1:100.000. Because nested PCR provided an amplicon of the 100.000-fold diluted test sample it was preferred over the qPCR (TaqMan). The advantage in using the nested PCR approach here is that positive samples can be directly sequenced and to generate a >1 kb amplicon which provides enough information for 16Sr subgroup assignment or '*Candidatus* (*Ca.*) Phytoplasma (P.)' species identification. Identification of the detected '*Ca.* P.' species and subgroups was performed by applying Sanger Sequencing on the amplicon generated by the nested PCR. For the two positive samples of the present study (Supplementary Figure S1), the base calling performed on the chromatograms did not result in any ambiguities, indicating the presence of a single phytoplasma strain in the positive samples. However, we are aware that other strains may be present at low concentrations and that they cannot be identified using the applied sequencing approach.

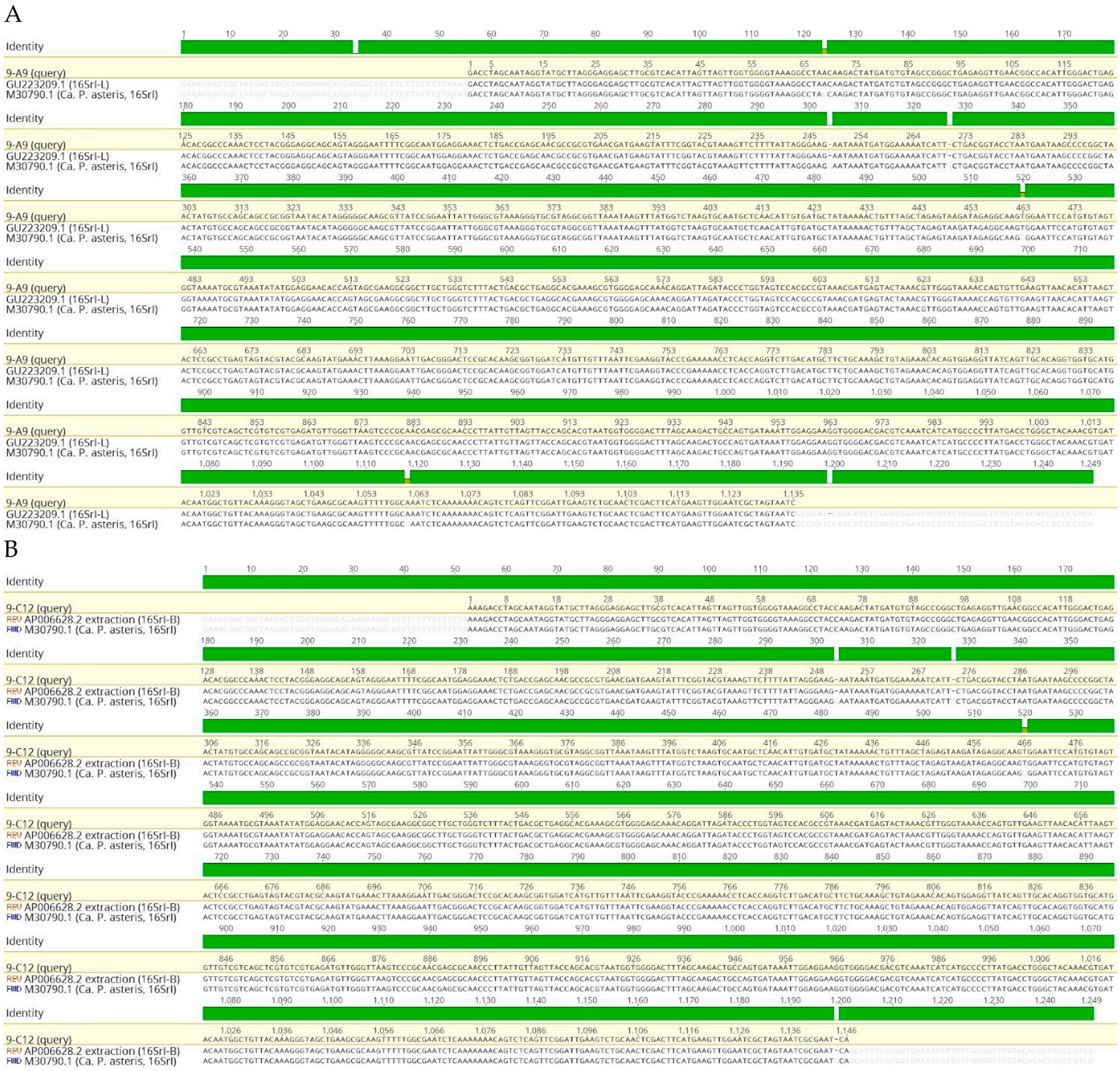

Figure S1. Sequence alignment of a 16S rRNA gene stretch amplified in samples 1209-F5-W (9-A9) and 1396-F8-M (9-C12). The 16S rRNA gene sequence stretch relevant for the phytoplasma subgroup identification using iPhyClassifier is depicted. In this alignment the amplicon sequence (query) from the nested PCR of sample 1209-F5-W (9-A9) and 1396-F8-M (9-C12) is compared to the reference sequence from '*Ca*. P. asteris' (M30790.1). Both samples share over 99 % 16S rRNA sequence similarity to the '*Ca*. P. asteris' reference strain, belonging to the 16SrI group. The sequence from sample 1209-F5-W (9-A9) is 100 % identical to the sequence of the 16SrI-L subgroup reference strain GU223209.1 (A). The sequence of 1396-F8-M (9-C12) is 100 % identical to the 16SrI-B subgroup reference strain AP006628.2 (B). Sequence alignment was performed using the software Geneious 11.1.5 (https://www.geneious.com). .
